# Interpregnancy Stability of the First-Trimester Uterine Artery Pulsatility Index: A Within-Woman Comparison with PAPP-A and Free β-hCG in 864 Women

**DOI:** 10.3390/diagnostics16193108

**Published:** 2026-09-24

**Authors:** Maria Cezara Mureșan, Marius Bogdan Mureșan, Dan Dumitrașcu Biriș, Zoran Laurențiu Popa

**Affiliations:** 1 Department of Obstetrics and Gynecology, Faculty of Medicine, Victor Babeș University of Medicine and Pharmacy, 300041 Timișoara, Romania; 2 Maternal-Fetal Medicine Center, 300331 Timișoara, Romania

**Keywords:** uterine artery doppler, pulsatility index, interpregnancy, spiral artery remodelling, PAPP-A, free β-hCG, intraclass correlation, variance components, first-trimester screening, regression to the mean

## Abstract

**Background/Objectives**: First-trimester screening combines a haemodynamic marker, the uterine artery pulsatility index (UtA-PI), with two serum markers, pregnancy-associated plasma protein A (PAPP-A) and free β-human chorionic gonadotropin (free β-hCG). For the serum markers, it is known that the value measured in one pregnancy correlates with the value measured in the same woman’s next pregnancy. For UtA-PI, only decreases in the mean value in second pregnancies have been reported. This study quantifies how much of a woman’s standardised UtA-PI persists from one pregnancy to the next and compares this persistence with that of the serum markers measured in the same woman. **Methods**: We performed a retrospective longitudinal analysis of the screening database of a single foetal medicine unit (August 2010–August 2026). Among 8925 eligible singleton pregnancies, 864 women were screened in two or more pregnancies (1757 pregnancies; 893 pairs of successive pregnancies; median interval 2.9 years, range 0.4–10.7 years). Marker values, expressed as multiples of the median (MoM) and log-transformed, were analysed with random-intercept models that split the total variation into a component between women and a component between the pregnancies of the same woman. The intraclass correlation coefficient (ICC), the fraction of the variation that lies between women, was estimated with bootstrap confidence intervals. **Results**: The ICC was 0.349 (95% confidence interval 0.280–0.412) for PAPP-A, 0.339 (0.274–0.397) for UtA-PI and 0.392 (0.290–0.478) for free β-hCG; that is, about one-third of the variation in each marker lay between women and about two-thirds between the pregnancies of the same woman. Adjustment for maternal age, body mass index, parity, smoking, gestational age, calendar time, biochemical analyser and examiner did not change the estimates (0.350 and 0.352). No weakening of the between-pregnancy correlation with a longer interval was detected (interaction *p* = 0.698 and 0.153), although statistical power was limited for the longest intervals. UtA-PI was lower in the next pregnancy by 6.3% on average (95% confidence interval 4.5–8.2%), whereas PAPP-A did not change. A previous PAPP-A below 0.4 MoM recurred in 17.4% of next pregnancies, and a previous UtA-PI above the 95th centile in 6%. **Conclusions**: Two distinct phenomena coexist in UtA-PI: a systematic fall at the population level in the next pregnancy, and a modest persistence of the individual value. Approximately one-third of the variation in UtA-PI reflects persistent differences between women—no more than for the serum markers—whereas two-thirds arises between the pregnancies of the same woman. A previous value carries some information about the next pregnancy but cannot replace a new measurement.

## 1. Introduction

First-trimester screening at 11–13 weeks combines two kinds of information about the developing placenta: a biochemical signal, represented by pregnancy-associated plasma protein A (PAPP-A) and free β-human chorionic gonadotropin (β-hCG), and a haemodynamic signal, represented by the uterine artery pulsatility index (UtA-PI). Both are expressed as multiples of the median (MoM) after adjustment for gestational age and for maternal characteristics such as weight, ethnicity, smoking, parity and mode of conception [1,2,3]. These standardised values enter the combined test for trisomy 21 [4] and the competing-risks model for pre-eclampsia (PE) [5,6,7], which now guides aspirin prophylaxis [8,9].

The MoM transformation removes systematic effects that can be modelled from a woman’s characteristics at the time of testing. Whatever remains is treated, in the screening algorithm, as pregnancy-specific. Yet a woman who has a low PAPP-A or a high UtA-PI in one pregnancy may tend to have similar values in the next because of stable constitutional factors that are not captured by the covariates, such as uterine vascular anatomy, endometrial and decidual biology, or genetic determinants of protein production. For serum markers, this has been known for almost three decades. Spencer described between-pregnancy correlation of second- and first-trimester markers [10,11,12], Larsen and Wald examined whether adjusting for the previous pregnancy improved Down syndrome screening [13,14], and Wright et al. reported correlations of 0.48 for PAPP-A and 0.44 for free β-hCG MoM in 8499 women with two consecutive pregnancies, and used them to modify the risk in the second pregnancy [15]. Illescas et al. also examined marker correlations in successive pregnancies [16]. Recurrence of low PAPP-A has been studied in relation to perinatal outcome [17], and the recurrence of PE itself is well documented [18,19].

For the haemodynamic marker, the situation is different. The reference ranges and MoM models for UtA-PI incorporate parity and a history of PE as covariates [3,20]. Serial measurements within a pregnancy have also been described [21,22], and the short-term reproducibility of the measurement is well characterised, with intra- and inter-observer intraclass correlation coefficients (ICC) of about 0.8–0.9 [23,24,25]. Additionally, operator drift and site dependence have been documented in routine practice [26,27,28]. Only one study, to our knowledge, has compared first-trimester UtA-PI between consecutive pregnancies of the same women: in a conference report on 920 women, Chatzakis et al. found a lower mean UtA-PI MoM in the second pregnancy (1.20 ± 0.29 versus 1.13 ± 0.30) and a positive association between the interpregnancy interval and the second-pregnancy value, which they interpreted as persistence of spiral artery remodelling from the first pregnancy that fades with time [29]. Their analysis addressed the population-level shift between pregnancies. It did not address the individual-level question—how much of a woman’s deviation from the median in one pregnancy is carried into the next—which requires partitioning the variance into between-woman and within-woman components. It also did not address whether the haemodynamic marker is more or less stable within women than the biochemical markers measured at the same visit. This parameter matters in two ways. Clinically, it determines how much a previous Doppler result should influence counselling and screening in a new pregnancy. Conceptually, comparing it with the biochemical markers in the same women asks whether the “uteroplacental signature” of the first trimester is a maternal trait or a pregnancy-level event.

The ICC from a random-intercept model quantifies this directly as the proportion of total variance attributable to between-woman differences [30]. Its complement pools everything that changes from one pregnancy to the next: pregnancy-specific placentation, changes in maternal state, technical variation and measurement error. We therefore aimed to (i) estimate the within-woman stability of first-trimester UtA-PI and PAPP-A MoM across successive pregnancies, using free β-hCG as a second biochemical comparator; (ii) test whether stability weakens with the interval between pregnancies or is explained by changes in maternal characteristics and technical factors; and (iii) describe the practical consequences—mean change, regression to the mean and recurrence of screen-positive values—for the interpretation of a previous result.

## 2. Materials and Methods

### 2.1. Study Design and Setting

This was a retrospective longitudinal study of routinely collected first-trimester screening data recorded in the ASTRAIA obstetric database (Astraia Software GmbH, Munich, Germany; version 30.1.2) of the Maternal-Fetal Medicine Center, Timișoara, Romania, a specialized foetal medicine centre, between August 2010 and August 2026; the database contains examinations from August 2009, and eligible pregnancies with both markers recorded were available from August 2010. Screening was performed as part of routine clinical care in women attending the centre for first-trimester assessment. The study was conducted in accordance with the Declaration of Helsinki and approved by the Ethics Committee of the Maternal-Fetal Medicine Center, Timișoara, Romania (framework approval No. 2/2026, 7 September 2026; initial approval No. 1/2026, 1 August 2026) for the retrospective secondary analysis of pseudonymised clinical data. The requirement for individual informed consent was waived by the Committee. Reporting follows the STROBE recommendations for cohort studies [31].

### 2.2. Measurements

Uterine artery Doppler was performed transabdominally at 11 + 0 to 13 + 6 weeks according to Fetal Medicine Foundation (FMF) protocol. Each uterine artery was sampled at the level of the internal os with an insonation angle below 30°, and the PI of the left and right arteries was averaged. Ultrasound examinations were performed using Voluson 730 Expert, Voluson E6, Voluson E10 and, during approximately the last seven years of the study period, Voluson Expert 22 systems (GE HealthCare, Zipf, Austria). First-trimester examinations, including nuchal translucency scanning and uterine artery Doppler, were performed by FMF-certified examiners according to FMF protocol. Maternal serum PAPP-A and free β-hCG were measured on the BRAHMS KRYPTOR (Thermo Fisher Scientific, Waltham, MA, USA), PerkinElmer DELFIA (Wallac Oy, Turku, Finland) and Roche platforms (Penzberg, Germany). Over the study period, the recorded platforms were BRAHMS KRYPTOR (73% of pregnancies), PerkinElmer DELFIA Xpress and Roche (the latter was used during approximately the last seven years), and the analyser used was recorded with each result. MoM values for PAPP-A, free β-hCG and UtA-PI were those calculated by ASTRAIA, which adjusts for gestational age by crown–rump length (CRL), maternal weight, ethnicity, smoking, parity, mode of conception and, for biochemistry, the analyser, according to the FMF reference models [1,2,3]. ASTRAIA has been used continuously at the centre since 2011 under a software agreement (examinations from 2009–2010 were entered into the same database), with official updates implemented as they became available. The MoM values analysed were those calculated at the time of each screening examination by the software version and reference model then in use, with biochemical MoM corrected for the analytical platform. No retrospective recalculation of MoM under a single uniform algorithm was performed, so the dataset reflects real-world longitudinal screening practice rather than a harmonised reanalysis. The dataset analysed was exported from ASTRAIA version 30.1.2 in August 2026 and contains examinations up to 6 August 2026. The left and right PI, the raw mean PI and the raw biochemical concentrations were also exported.

### 2.3. Cohort Construction and Pseudonymisation

The export contained 20,062 examination records. Eligible pregnancies were singleton, with CRL 45–84 mm and both PAPP-A MoM (0.01–20) and UtA-PI MoM (0.1–10) recorded. One record was included per pregnancy, yielding 8962 pregnancies in 8045 women. Records were linked at the woman level by the database patient identifier and at the pregnancy level by the patient–case–foetus key; both were replaced by salted one-way hashes before analysis, and names, dates of birth, contact details and examination dates were not exported. Examiners were coded anonymously. Because a woman with two pregnancy keys on the same examination date may represent an administrative duplicate rather than two pregnancies, all records of the 13 women with such same-day groups (37 pregnancies) were excluded conservatively, leaving 8925 pregnancies in 8032 women. The longitudinal cohort comprised the 864 women with at least two eligible pregnancies. Pregnancy order was defined by the chronology of the first-trimester examinations; the interval between successive pregnancies was the time between the two examinations (i.e., between comparable gestational ages), which does not equal the delivery-to-conception interval and cannot account for pregnancies managed elsewhere or ending before 11 weeks.

### 2.4. Statistical Analysis

In plain terms, the analysis asks: of all the variation in a marker across the 1757 pregnancies, what fraction is due to lasting differences between women, and what fraction to differences between the pregnancies of the same woman? The first fraction is the intraclass correlation coefficient (ICC): an ICC of 1 would mean that a woman’s value is identical in every pregnancy, and an ICC of 0 would mean that her previous value tells nothing about the next. MoM values were analysed on the natural logarithmic scale. For each marker, a linear mixed model with a random intercept for woman was fitted by restricted maximum likelihood, y_ij = β_0_ + b_i + ε_ij, where b_i ~ N(0, σ^2^_b) is the woman effect and ε_ij ~ N(0, σ^2^_w) is the pregnancy-specific residual. The ICC, σ^2^_b/(σ^2^_b + σ^2^_w), was the primary estimand. For descriptive context, ICC values were compared with the conventional thresholds of Koo and Li [30], while recognising that these thresholds were developed for reliability studies rather than for biological stability across pregnancies. Confidence intervals were obtained by non-parametric bootstrap with 2000 resamples of women (percentile method); the point estimates were checked against the one-way ANOVA estimator. Body mass index (BMI), a characteristic known to be highly stable within women, was analysed in the same way as a positive control for the method. The raw (unstandardised) mean UtA-PI was also analysed to determine whether the MoM transformation created or removed clustering.

A covariate-adjusted ICC was estimated from the residuals of a regression of ln(MoM) on maternal age, BMI, parity, current smoking, CRL, calendar year (continuous), analyser and examiner, to test whether the clustering was explained by measured characteristics that changed between pregnancies or by technical drift. Adjacent pregnancies of the same woman formed pairs; the mean log change (second minus first) with woman-clustered standard errors was exponentiated to a geometric mean ratio. Pearson correlations between adjacent pregnancies, with woman-level bootstrap CIs, were computed overall and within strata of interval (<2, 2–4, >4 years), analyser concordance and examiner concordance. To allow direct comparison with the report of Chatzakis et al. [29], the mean UtA-PI MoM (±SD) of the first and second pregnancy and the paired difference were also computed on the MoM scale, and the second-pregnancy UtA-PI MoM was regressed at the interpregnancy interval. Interval dependence of persistence was tested formally by the interaction between the (centred) previous value and the (centred) interval in a regression of the current value that also included changes in BMI and smoking and the concordance indicators. Signed changes were regressed on changes in BMI, changes in smoking, interval, parity and the concordance indicators. As an internal reference for UtA-PI, the correlation between the left and right ln PI at the same examination was computed in the eligible cohort. Regression to the mean was described as the median (interquartile range, IQR) of the next-pregnancy MoM within quintiles of the previous value. Recurrence of screen-positive values (PAPP-A < 0.4 MoM, <0.5 MoM and <5th centile; UtA-PI > 90th and >95th centile of the eligible cohort) was expressed as the proportion positive in the next pregnancy given a positive previous result, the corresponding proportion after a negative result, their ratio, and Cohen’s κ. Sensitivity analyses repeated the ICC after restricting to the first two pregnancies, to women with exactly two pregnancies, to women without any interval below nine months, to recorded gestational age 77–97 days, to spontaneous conceptions, to the most frequent examiner, to the most frequent analyser, after trimming values outside the 1st–99th centiles, and including women with a single pregnancy. Covariate-adjusted analyses used complete cases. In the longitudinal cohort, the two principal markers, BMI, parity, CRL, smoking, analyser and examiner were complete; maternal age was missing for both pregnancies of one woman (2 of 1757 records) and free β-hCG for one pregnancy, so the adjusted models included 1755 pregnancies (1754 for free β-hCG). Analyses used Python 3.11 with pandas 3.0 and statsmodels 0.14; two-sided *p*-values are reported without adjustment except where stated.

## 3. Results

### 3.1. Cohort

The longitudinal cohort comprised 864 women and 1757 pregnancies (836 women with two, 27 with three and 1 with four eligible pregnancies), providing 893 adjacent pairs. The median interval between first-trimester examinations was 2.89 years (IQR 2.03–4.31; range 0.44–10.71); 217 pairs were separated by less than two years, 416 by two to four years and 260 by more than four years, and 11 pairs by less than nine months. The same analyser was used in 585 pairs (66%) and the same examiner in 823 (92%); one examiner performed 95.0% of first and 90.9% of second examinations. Table 1 describes the first and second pregnancy of each woman and, for comparison, the 7168 women with a single eligible pregnancy. Between the first and the second pregnancy the women were on average three years older, had gained about one BMI unit and had become parous; the median PAPP-A and free β-hCG MoM were essentially unchanged, whereas the median UtA-PI MoM fell from 1.082 to 1.024. Women who returned for a second pregnancy were younger, leaner, more often nulliparous and less often smokers at their first examination than women with a single eligible pregnancy, and they had been screened earlier in the study period.

### 3.2. Within-Woman Stability

The ICC was 0.349 (95% CI 0.280–0.412) for PAPP-A, 0.339 (95% CI 0.274–0.397) for UtA-PI and 0.392 (95% CI 0.290–0.478) for free β-hCG (Table 2). In other words, 65.1%, 66.1% and 60.8% of the variance in the three standardised markers lay within women, between pregnancies. The three confidence intervals overlapped almost entirely. The estimate for the raw mean UtA-PI (0.334 (95% CI 0.259–0.395)) was the same as for the MoM, showing that the standardisation neither created nor removed the clustering; this comparison is only approximate, because the raw PI is not adjusted for gestational age, which is itself a source of within-woman variability. The method itself detected strong stability where it exists: the ICC for BMI in the same women and pregnancies was 0.825 (95% CI 0.796–0.849). Adjustment for maternal age, BMI, parity, smoking, CRL, calendar time, analyser and examiner left the residual ICCs unchanged, at 0.350 (0.279–0.415) for PAPP-A, 0.352 (0.287–0.418) for UtA-PI and 0.383 (0.282–0.470) for free β-hCG; the between-woman component was therefore not an artefact of measured maternal characteristics or of technical drift.

### 3.3. Mean Change, Persistence and Regression to the Mean

Between adjacent pregnancies, PAPP-A did not change on average (geometric mean ratio 1.014, 95% CI 0.972–1.058; *p* = 0.51), nor did free β-hCG (0.976, 0.935–1.019; *p* = 0.28), whereas UtA-PI MoM was lower in the next pregnancy by a geometric mean of 6.3% (ratio 0.937, 95% CI 0.918–0.955; *p* < 0.001). This is the unadjusted paired estimate; because 92% of women were nulliparous at the first and 96% parous at the second eligible pregnancy, the change between pregnancies and the change in parity are almost completely confounded in this cohort, and the estimate should not be read as independent of parity. On the MoM scale, the mean (±SD) UtA-PI was 1.12 ± 0.29 in the first and 1.05 ± 0.27 in the second pregnancy (paired difference 0.073 MoM, 95% CI 0.051–0.095; *p* < 0.001). Figure 1 shows the first two pregnancies of each woman. The slope of the second on the first value was 0.348 for PAPP-A and about the same for UtA-PI, i.e., only about one-third of a woman’s deviation from the population median in one pregnancy was, on average, carried into the next. The Bland–Altman limits of agreement (mean ± 1.96 SD of the paired difference) spanned ±1.3 ln units for PAPP-A—a 3.6-fold range—and −0.66 to +0.53 ln units for UtA-PI [32].

This regression to the mean [33]—the expected consequence of a correlation of about 0.35—is shown by quintile of the previous value in Figure 2 and Table 3. Women in the lowest PAPP-A quintile (median 0.461 MoM) had a median of 0.723 MoM (IQR 0.522–1.014) in the next pregnancy; those in the highest UtA-PI quintile (median 1.54 MoM) had a median of 1.134 MoM (0.958–1.394). The ordering of the quintiles was preserved for both markers—a woman’s previous quintile remained informative—but the spread within each quintile was wide.

### 3.4. Interval, Maternal Change and Technical Factors

No weakening of the correlation between adjacent pregnancies with the time between them was detected (Figure 3), although only 260 of the 893 pairs were separated by more than four years and the confidence intervals of the interaction terms were wide. For PAPP-A, r was 0.334, 0.358 and 0.349 for intervals of <2, 2–4 and >4 years; for UtA-PI it was 0.385, 0.335 and 0.403; and for free β-hCG, 0.486, 0.361 and 0.385. The interaction between the previous value and the interval was 0.007 per year (95% CI −0.030 to 0.045; *p* = 0.698) for PAPP-A, 0.031 (−0.011 to 0.073; *p* = 0.153) for UtA-PI and 0.003 (−0.033 to 0.039; *p* = 0.872) for free β-hCG. The second-pregnancy UtA-PI MoM rose slightly with the interval (mean 1.02, 1.05 and 1.07 MoM for <2, 2–4 and >4 years; 0.005 MoM per year, 95% CI −0.005 to 0.015; *p* = 0.328), and the standard deviation of the paired difference was constant across interval strata (PAPP-A 0.666, 0.658, 0.623; UtA-PI 0.284, 0.316, 0.285).

Correlations were also similar whether or not the two pregnancies were tested on the same analyser (PAPP-A 0.342 vs 0.355) or scanned by the same examiner (UtA-PI 0.356 vs 0.453; only 70 pairs had different examiners). Changes in BMI and in smoking between pregnancies, the interval and parity explained 2% and 1% of the variance of the signed change in PAPP-A and UtA-PI, respectively. None of these terms was associated with the change (all *p* > 0.1), with the exception of a nominal association between examiner concordance and the signed change (PAPP-A β = −0.24, *p* = 0.003; UtA-PI β = 0.09, *p* = 0.019). However, this was confounded with the calendar period and disappeared once calendar terms were included.

As a contextual benchmark, the correlation between the left and right ln UtA-PI measured at the same examination was 0.393 in the 8916 eligible pregnancies with both values (0.39 in the longitudinal cohort), i.e., of the same order as the correlation between the mean UtA-PI of two pregnancies years apart (0.366).

### 3.5. Recurrence of Screen-Positive Values

Table 4 gives the recurrence of the categorical results that are used in practice. A PAPP-A < 0.4 MoM recurred in 8 of 46 next pregnancies (17.4%), compared with 4.8% after a previous PAPP-A ≥ 0.4 MoM (ratio 3.59); a UtA-PI above the 95th centile (1.62 MoM) recurred in 3 of 50 (6%) versus 3.1% (ratio 1.95), and above the 90th centile in 17.9% versus 6.3% (ratio 2.85). These ratios rest on small numbers of events—only three recurrences for the 95th-centile UtA-PI threshold—and are therefore imprecise; they should be read as qualitative indications rather than as stable estimates. Agreement beyond chance was slight for all thresholds (κ 0.04–0.16). Thus, a previous screen-positive result raised the probability of a positive result in the next pregnancy two- to four-fold, but in absolute terms, three of four women with a previous low PAPP-A and more than nine of ten women with a previous high UtA-PI were screen-negative on that marker next time.

### 3.6. Sensitivity Analyses

The ICCs were stable across all restrictions (Table 5), ranging between 0.31 and 0.39 for PAPP-A and between 0.33 and 0.36 for UtA-PI, including the analysis restricted to the principal examiner (0.345 for UtA-PI) and to the principal analyser (0.307 for PAPP-A). Including the 7168 women with a single pregnancy, which adds information on the total variance, gave 0.355 and 0.361.

## 4. Discussion

### 4.1. Principal Findings

In 864 women screened in two or more pregnancies at the same unit, two distinct phenomena were observed in the first-trimester UtA-PI. At the population level, the marker was systematically lower at the next pregnancy, by about 6%, whereas the serum markers showed no shift. At the individual level, a woman’s rank persisted only moderately. Approximately one-third of the residual variation of UtA-PI MoM was attributable to persistent between-woman differences and two-thirds arose within women between pregnancies—a proportion no higher than that of PAPP-A and free β-hCG measured at the same visits. The individual-level persistence was not explained by maternal age, BMI, parity, smoking, calendar time, analyser or examiner, showed no detectable decline over intervals of up to ten years (with limited power beyond four years), and was the same for the raw and the standardised UtA-PI. In practical terms, a woman’s previous result predicts her next one only partially. About one-third of the deviation from the median is carried forward, three-quarters of low PAPP-A results and more than 90% of high UtA-PI results are not repeated, and the wide within-quintile spread means that a new measurement is indispensable.

### 4.2. Comparison with Previous Studies

For the serum markers, our estimates extend a consistent literature. Spencer [10,11,12] and Larsen et al. [13] first described between-pregnancy correlation of screening markers, Wald et al. [14] showed that adjusting for a previous pregnancy yields only a small gain in Down syndrome screening performance because that correlation is modest, and Wright et al. [15] reported correlations of 0.48 for PAPP-A and 0.44 for free β-hCG in 8499 women, somewhat higher than our 0.35 and 0.40. The difference is compatible with differences in population, laboratory setting, selection into repeat screening and, in our data, changes of analyser over a 16-year period. Analyser concordance within pairs did not affect the correlation within our study, but this internal check does not address why the overall correlation might differ from that of another cohort. The ICC and the pairwise correlation are also different estimands. The ICC uses all pregnancies of each woman and is bounded by the same variance components, and in our data, the two coincided. Zafman et al. [17] observed normalisation of a previously low PAPP-A in 74% of subsequent pregnancies, in close agreement with the 76–83% we found for the <0.5 and <0.4 MoM thresholds.

For UtA-PI, the population-level result replicates the only previous between-pregnancy comparison with remarkable fidelity. Chatzakis et al. reported mean UtA-PI MoM of 1.20 ± 0.29 in the first and 1.13 ± 0.30 in the second pregnancy of 920 women (difference 0.07, 95% CI 0.03–0.10) [29]. We found 1.12 ± 0.29 and 1.05 ± 0.27 in 864 women (difference 0.07, 95% CI 0.05–0.10). These values reflect relative decreases of 5.8% in their data and 6.3% in ours. They also observed that the second-pregnancy UtA-PI increased with the interpregnancy interval (0.004 MoM per year, 95% CI 0.002–0.005), which they interpreted as a fading of the vascular remodelling left by the first pregnancy; our point estimate was the same (0.005 MoM per year) with a wider interval that included zero. The decline is consistent with the lower UtA-PI of parous women in the FMF model [3] (although not all studies found a parity effect [34]) and with the lower risk of PE in second pregnancies without a history of PE [18]. It also indicates that the MoM model, which in our software, could not be confirmed to include parity for every version, does not remove this effect completely. Order, parity, age and calendar time advance together in a longitudinal cohort. Almost every woman was nulliparous at her first and parous at her second eligible pregnancy, so the 6.3% decline is, by construction, also a nulliparous-to-parous comparison, and its causal attribution to pregnancy order, parity or age is not possible in this design. (The covariate-adjusted ICC includes parity, but that adjustment concerns the persistence of the individual value, not the mean decline.) The same confounding also affects the persistence estimate. If part of the change in UtA-PI with parity is a directional, near-deterministic physiological shift—for example, persistent spiral artery remodelling after a first pregnancy—rather than random fluctuation, then some of what is labelled within-woman variance reflects that systematic shift, and the ICC would understate the stability of the individual value net of parity. Because almost every woman changed parity status between her two eligible pregnancies, the two components cannot be separated with the present data; the estimates of persistence should therefore be read as applying to the transition from a first to a subsequent pregnancy as observed in routine screening, not as a parity-independent constant.

### 4.3. Interpretation

What the present study adds is individual-level analysis, which the population-level comparison cannot provide. A systematic shift and persistent individual ranking address different questions. Regarding the latter, persistence is modest: the ICC of 0.34 would fall in the lowest category of conventional reliability scales, such as those proposed by Koo and Li [30]. However, these thresholds were developed for test–retest and inter-rater reliability, and the label here refers to the biological stability of a value across pregnancies years apart, not to the quality of the measurement. The between-pregnancy correlation of 0.37 implies that only about a third of a woman’s deviation is carried forward, and a previous UtA-PI above the 95th centile was followed by a value above that centile in only 6% of next pregnancies. This also resolves an apparent tension with Chatzakis et al. [29]: the interval was associated in their data (and, with less precision, in ours) with the *level* of UtA-PI in the second pregnancy, whereas we found no association between the interval and the *strength of persistence* of the individual value. These are different statistical questions—one concerns the mean, the other the correlation—and both can be true. The existing Doppler literature otherwise describes short-term reproducibility, with intra- and inter-observer ICCs of about 0.90 and 0.84 in a recent meta-analysis [25]. Measurable operator-level drift has also been reported in routine screening [26]. By comparison, the between-pregnancy ICC of 0.34 is far below these short-term values. The difference is consistent with an important contribution of biological variation between pregnancies, but the within-woman component cannot be partitioned into measurement error and placental biology with the present design, and we do not attempt to quantify their respective shares. The left–right correlation at a single examination (0.39) offers a contextual benchmark of the same order, although it should not be read mechanistically. The two arteries differ for reasons of placental laterality as well as measurement error, and the two comparisons are not equivalent.

The similarity between markers that measure different things by different methods invites a speculative interpretation, which we offer as a hypothesis consistent with the data rather than as a conclusion drawn from them. PAPP-A and free β-hCG are trophoblast products assayed in serum; UtA-PI is a Doppler index of uterine vascular impedance obtained by an operator. If between-pregnancy stability was mainly a property of the method—assay batch, operator habit, sampling site—one would expect the biochemical and the haemodynamic markers to differ in their ICCs and the correlations to depend on analyser or examiner concordance. Neither was the case. If stability was mainly a slowly changing maternal state, one would expect it to erode over longer intervals and to track changes in BMI or smoking; it did neither. The remarkable similarity between the ICCs of a vascular Doppler index and two trophoblast products is consistent with, although it does not prove, a common maternal constitutional component—vascular anatomy, decidual biology, genetic regulation of placental protein expression—underlying roughly one-third of the residual variation. The remaining two-thirds encompass pregnancy-specific placental biology together with technical and measurement variation that the present design cannot separate. This is consistent with the recurrence pattern of PE itself, which is strongly elevated after an affected pregnancy but far from deterministic [18,19].

The findings have three practical consequences. First, the previous results cannot substitute for measurement: the within-quintile spread and the low recurrence of screen-positive values are such that a woman whose PAPP-A was 0.3 MoM has a median of about 0.7 MoM next time, with a quarter above 1.0. Second, the previous results are nevertheless informative and could be used quantitatively, as Wright et al. proposed for biochemistry [15]. Our correlations provide, for the first time, the parameter that such an adjustment would require for UtA-PI, and they show that it is of the same order as for the serum markers. Third, the data allow counselling in probabilistic terms: a previous UtA-PI above the 95th centile roughly doubles the chance of a repeat high value, but the absolute probability remains below 10%, and a previous low PAPP-A raises the chance of recurrence about three-fold, to roughly one in six for the 0.4 MoM threshold and one in four for 0.5 MoM. Whether adding the previous pregnancy’s markers improves prediction of PE or foetal growth restriction beyond current measurements and maternal history is a separate question that requires outcome data and is not answered here.

### 4.4. Strengths and Limitations

Strengths include the size of the longitudinal cohort, which is comparable to the only previous between-pregnancy Doppler series [29] and to the biochemical series; the use of all pregnancies per woman in the variance-component model; woman-level bootstrap inference; a within-cohort positive control (BMI); a second biochemical comparator (free β-hCG); an internal Doppler reference (left–right correlation); and extensive sensitivity analyses. A single unit with one principal examiner and one principal analyser limits technical heterogeneity but also limits generalisability; the correlation estimates are, however, unchanged in the subsets with different examiners or analysers.

Several limitations should be noted. Eligibility required both markers, so the cohort reflects women who completed combined screening, and repeat attendance was selective. Women who returned were younger, leaner and less often smokers at their first visit. Pregnancies managed elsewhere, or ending before 11 weeks, are invisible, so “successive” means successive in the database and the interval was measured between screening examinations rather than from delivery to conception. The MoM values were those of the clinical software; we could not reconstruct the exact reference models used in each year, although the identical ICC for raw and standardised UtA-PI indicates that this does not affect the conclusions. The residual variance combines pregnancy-specific biology with measurement error, which we could not separate directly; the left–right correlation gives only an indirect bound. Outcome data were incomplete and were not analysed. Review of the six women whose records showed a decrease in recorded parity between pregnancies (four) or a change in recorded height of more than 5 cm (two) identified inconsistencies in the maternal history fields (for example, a recorded parity lower than in the previous record despite an intervening live birth, or a height entered in metres). The marker values themselves were not affected, the women were retained, and excluding them left the ICCs unchanged (0.354 for PAPP-A, 0.336 for UtA-PI and 0.391 for free β-hCG in 858 women). Calendar year entered the adjusted ICC as a continuous linear term. Discrete changes tied to specific software versions, reference-model updates or platform transitions may not be fully captured by such a term and remain a residual source of within-woman variation, although the sensitivity analyses restricted to a single analyser or to the principal examiner gave similar estimates. Finally, the secondary analyses were numerous and should be regarded as descriptive.

## 5. Conclusions

The first-trimester UtA-PI shows two separable phenomena across successive pregnancies: a systematic fall of about 6% in the next pregnancy, consistent with the only previous report, and a modest persistence of individual values, quantified here for the first time. Approximately one-third of the residual variation is attributable to persistent between-woman differences, while two-thirds arises within women between pregnancies. This persistence is no greater than that of PAPP-A and free β-hCG measured in the same women, showed no detectable dependence on the interval between pregnancies (with limited power for the longest intervals), and is not explained by measured maternal or technical factors. Previous results therefore carry real but limited information: they modify the expectation for the next pregnancy and can be expressed as recurrence probabilities for counselling, but they cannot replace measurement, and screening should be repeated in full in every pregnancy.

## Figures and Tables

**Figure 1:**
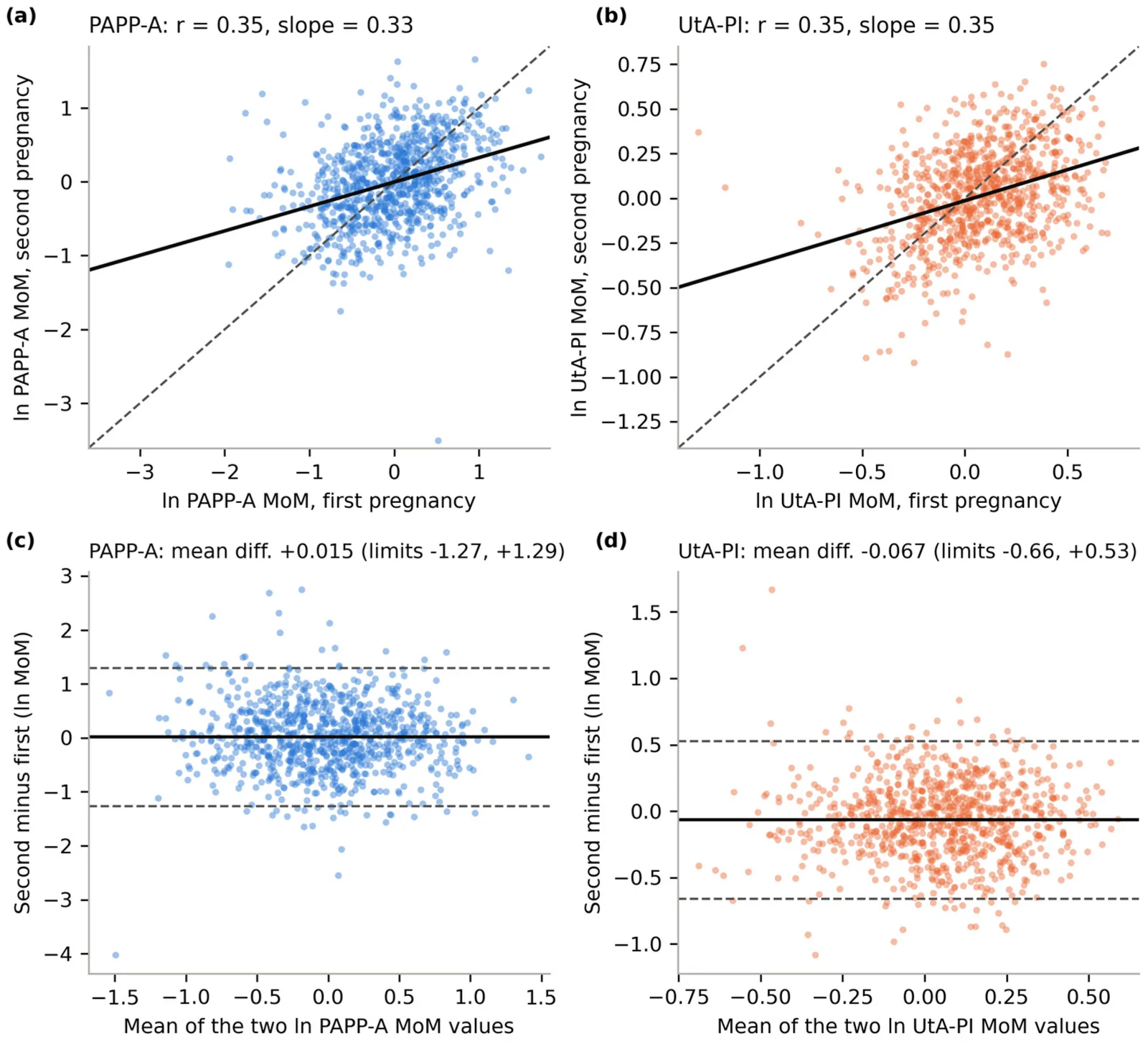
Persistence and paired dispersion of PAPP-A (blue) and UtA-PI (orange) in the first two eligible pregnancies of 864 women. (**a**,**b**) ln MoM in the second against the first pregnancy; dashed line, identity; solid line, least-squares fit. (**c**,**d**) Difference (second minus first) against the mean of the pair; solid line, mean difference; dashed lines, mean ± 1.96 SD.

**Figure 2:**
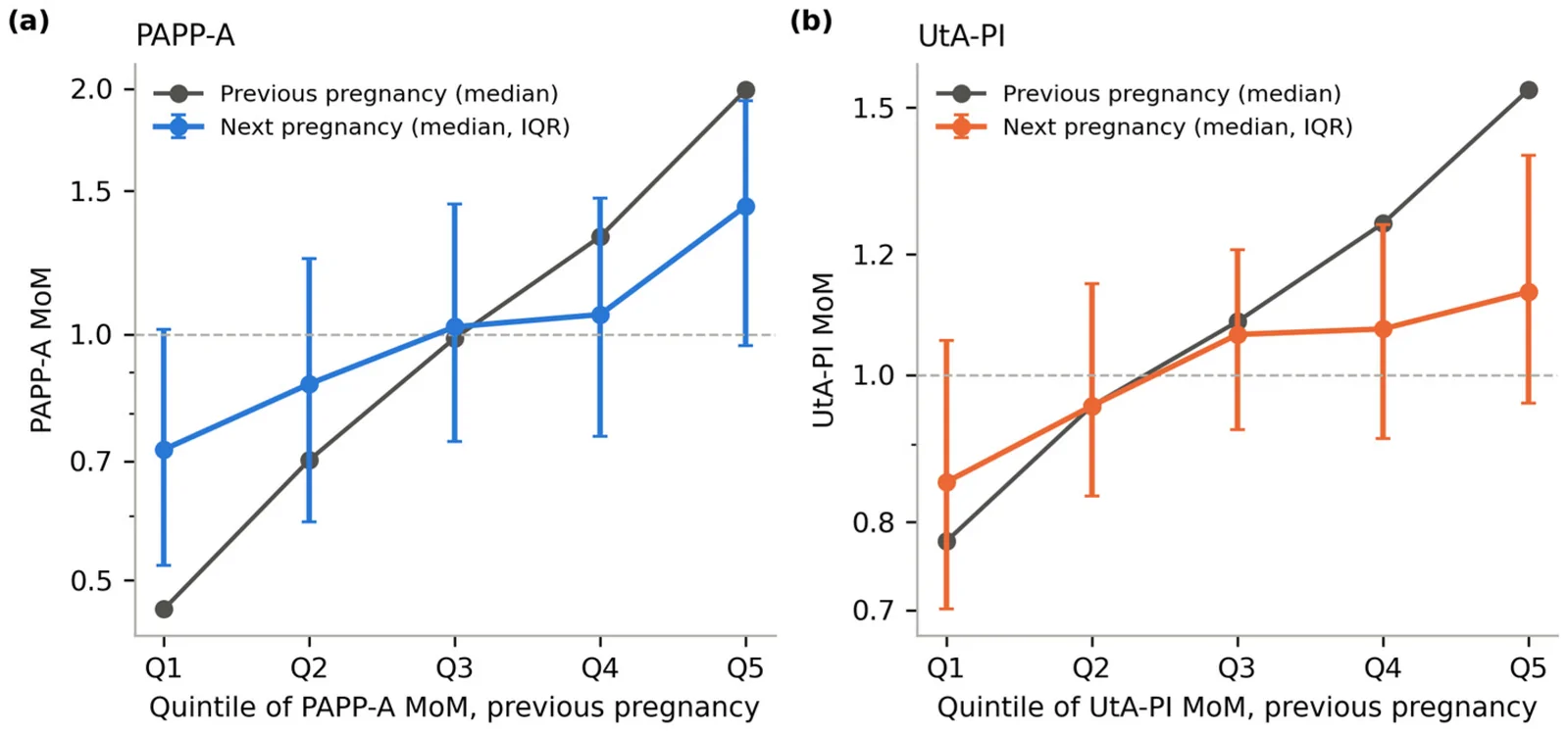
Regression to the mean. Median MoM in the previous pregnancy (grey) and median with interquartile range in the next pregnancy (colour) by quintile of the previous value, for (**a**) PAPP-A and (**b**) UtA-PI; 893 adjacent pairs. The horizontal dashed line marks 1.0 MoM.

**Figure 3:**
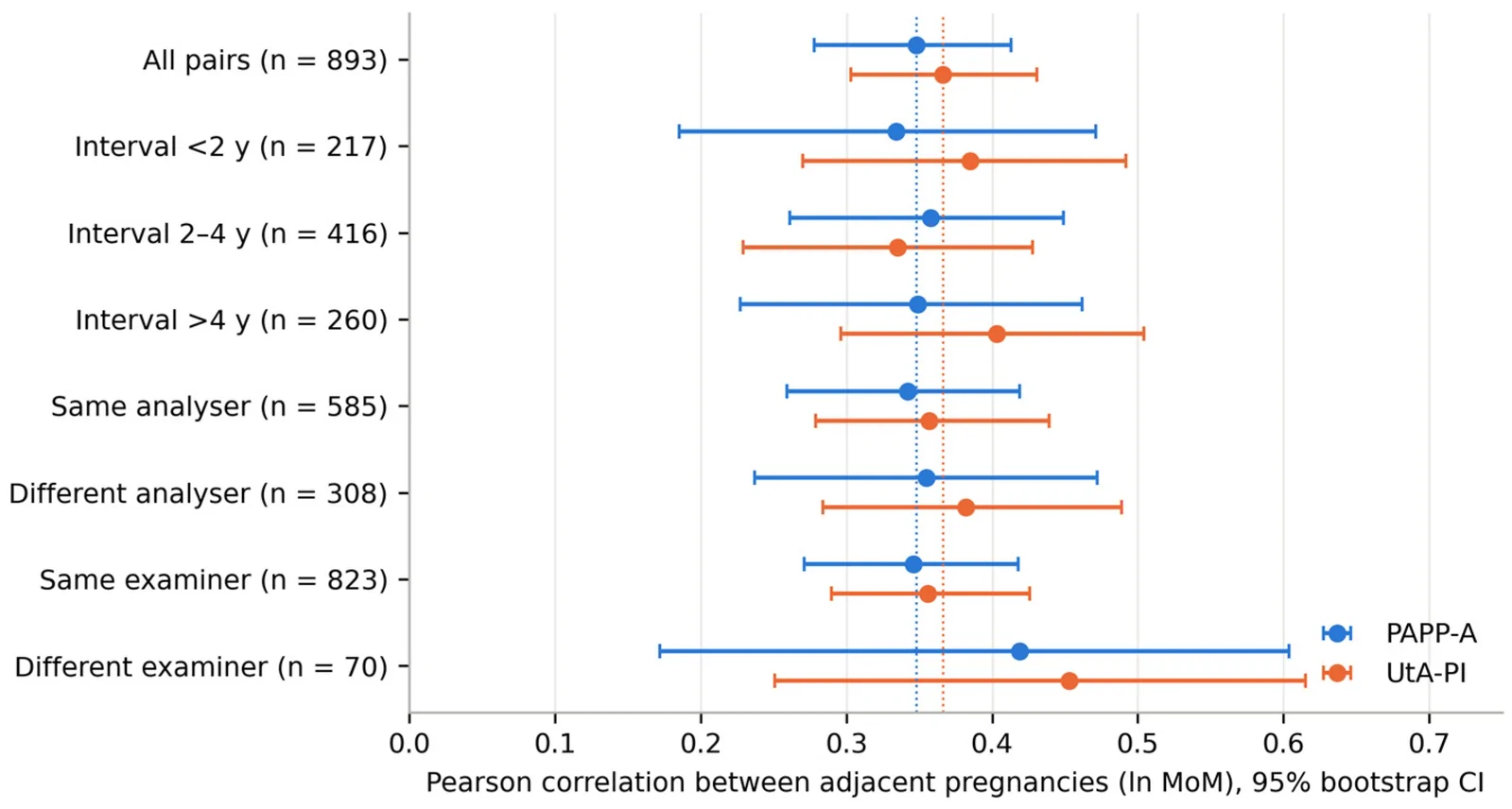
Pearson correlation between adjacent pregnancies (ln MoM) with 95% woman-level bootstrap confidence intervals, overall and by interval between examinations, analyser concordance and examiner concordance. Dotted lines mark the overall estimates.

**Table 1:** Characteristics of the first and second eligible pregnancies of the 864 women in the longitudinal cohort and of women with a single eligible pregnancy.

Characteristic	First Pregnancy (*n* = 864)	Second Pregnancy (*n* = 864)	Single-Pregnancy Women (*n* = 7168)
Maternal age, years	29.65 (27.01–32.19)	32.90 (30.22–35.70)	31.42 (28.17–35.16)
Body mass index, kg/m^2^	21.75 (19.84–24.22)	22.85 (20.62–25.71)	22.66 (20.45–25.82)
Nulliparous, *n* (%)	792 (91.7)	34 (3.9)	5002 (69.8)
Current smoker, *n* (%)	77 (8.9)	71 (8.2)	1056 (14.7)
Stopped smoking, *n* (%)	46 (5.3)	23 (2.7)	236 (3.3)
Spontaneous conception, *n* (%)	769 (89.0)	834 (96.5)	6604 (92.1)
Gestational age at examination, days	90.00 (87.00–93.00)	91.00 (88.00–94.00)	90.00 (87.00–93.00)
Crown–rump length, mm	65.50 (60.80–70.72)	67.40 (61.77–73.22)	66.30 (60.80–72.30)
PAPP-A, MoM	1.003 (0.649–1.433)	0.984 (0.683–1.459)	0.976 (0.670–1.428)
Free β-hCG, MoM	1.113 (0.749–1.633)	1.052 (0.745–1.607)	1.117 (0.753–1.656)
UtA-PI, MoM	1.082 (0.918–1.306)	1.024 (0.850–1.208)	1.052 (0.876–1.264)
BRAHMS Kryptor analyser, *n* (%)	653 (75.6)	612 (70.8)	5210 (72.7)
Principal examiner, *n* (%)	821 (95.0)	785 (90.9)	5959 (83.1)
Calendar year of examination	2016 (2013–2019)	2020 (2017–2023)	2020 (2016–2023)

Values are median (interquartile range) unless stated. In the longitudinal cohort, maternal age was missing for one woman (both of her pregnancies) and no other displayed variable was missing; in the single-pregnancy group, maternal age was missing for one woman and BMI for 15 women. Pregnancy order refers to eligible examinations in the database. MoM, multiples of the median; UtA-PI, uterine artery pulsatility index.

**Table 2:** Variance components and intraclass correlation coefficients (ICCs) of first-trimester markers across successive pregnancies (ln MoM scale; random-intercept models, REML).

Marker	Pregnancies/Women	Between-Woman Variance	Within-Woman Variance	Within-Woman Share, %	ICC (95% CI)
PAPP-A MoM	1757/864	0.1135	0.2119	65.1	0.349 (0.280–0.412)
UtA-PI MoM	1757/864	0.0243	0.0475	66.1	0.339 (0.274–0.397)
Free β-hCG MoM	1756/864	0.1445	0.2240	60.8	0.392 (0.290–0.478)
UtA-PI (raw mean PI)	1633/—	0.0254	0.0505	66.6	0.334 (0.259–0.395)
BMI (reference)	1757/864	12.3283	2.6165	17.5	0.825 (0.796–0.849)
PAPP-A MoM (adjusted)	1755/864	0.1114	0.2067	65.0	0.350 (0.279–0.415)
UtA-PI MoM (adjusted)	1755/864	0.0242	0.0446	64.8	0.352 (0.287–0.418)
Free β-hCG MoM (adjusted)	1754/864	0.1391	0.2240	61.7	0.383 (0.282–0.470)

Confidence intervals are woman-level percentile bootstrap intervals (2000 resamples for unadjusted, 1000 for adjusted estimates). Adjusted estimates are ICCs of the residuals after regression on maternal age, body mass index, parity, current smoking, crown–rump length, calendar year, analyser and examiner (complete cases). BMI is shown as a positive control for a characteristic known to be stable within women. Raw mean UtA-PI was available for 1633 pregnancies (women with ≥2 available values).

**Table 3:** Regression to the mean: marker level in the next pregnancy according to the quintile of the previous pregnancy (893 adjacent pairs).

Quintile of Previous Value	*n*	PAPP-A Previous, Median MoM	PAPP-A Next, Median (IQR) MoM	UtA-PI Previous, Median MoM	UtA-PI Next, Median (IQR) MoM
Q1 (lowest)	179/179	0.46	0.72 (0.52–1.01)	0.78	0.85 (0.70–1.05)
Q2	178/178	0.70	0.87 (0.59–1.24)	0.95	0.95 (0.83–1.15)
Q3	180/181	0.99	1.02 (0.74–1.45)	1.08	1.06 (0.92–1.21)
Q4	177/176	1.32	1.06 (0.75–1.47)	1.26	1.07 (0.91–1.26)
Q5 (highest)	179/179	1.99	1.44 (0.97–1.93)	1.54	1.13 (0.96–1.39)

Quintiles were defined separately for each marker on the previous-pregnancy ln MoM. IQR, interquartile range.

**Table 4:** Recurrence of screen-positive marker values in the next pregnancy (893 adjacent pairs).

Criterion	Positive in Previous Pregnancy, *n*	Positive in Both, *n*	Recurrence, %	Positive After Negative, %	Ratio	Cohen’s κ
PAPP-A < 0.4 MoM	46	8	17.4	4.8	3.59	0.12
PAPP-A < 0.5 MoM	111	27	24.3	9.0	2.72	0.16
PAPP-A < 5th centile (0.37 MoM)	41	6	14.6	3.6	4.02	0.12
UtA-PI > 90th centile (1.48 MoM)	112	20	17.9	6.3	2.85	0.14
UtA-PI > 95th centile (1.62 MoM)	50	3	6.0	3.1	1.95	0.04

Centiles were calculated from the 8925 eligible pregnancies. Ratio is the recurrence divided by the proportion positive after a negative previous result.

**Table 5:** Sensitivity of the ICC (ln MoM) to analytic restrictions.

Restriction	Pregnancies/Women	PAPP-A	UtA-PI	Free β-hCG
First two pregnancies only	1728/864	0.347	0.331	0.390
Women with exactly two pregnancies	1672/836	0.344	0.332	0.391
Exclude women with any interval <9 months	1735/853	0.359	0.339	0.391
Recorded gestational age 77–97 days	1611/794	0.344	0.330	0.392
Spontaneous conceptions only	1561/770	0.361	0.346	0.391
Most frequent examiner only (EX01)	1573/773	0.348	0.345	0.384
BRAHMS Kryptor analyser only	1021/503	0.307	0.327	0.452
Trim outside 1st–99th centile (both markers)	1658/815	0.386	0.356	0.448
All women passing same-day filter (incl. single observations)	8925/8032	0.355	0.361	0.391

Row restrictions retain women with at least two remaining pregnancies, except the last row. Trimming uses the 1st–99th centiles of the eligible cohort for both markers simultaneously.

## Data Availability

The pseudonymised dataset is held by the Maternal-Fetal Medicine Center, Timișoara, and is not publicly available because of patient confidentiality restrictions; aggregate data supporting the findings are available from the corresponding author on reasonable request. The analysis code (art1_build.py for cohort construction and pseudonymisation; art1_final.py for the statistical analyses and figures) and the pseudonymised analytic dataset structure are available from the corresponding author on reasonable request.
